# Monosodium Glutamate (MSG)-Induced Male Reproductive Dysfunction: A Mini Review

**DOI:** 10.3390/toxics8010007

**Published:** 2020-01-22

**Authors:** Omowumi T. Kayode, Damilare E. Rotimi, Abolanle A. A. Kayode, Tomilola D. Olaolu, Oluyomi S. Adeyemi

**Affiliations:** 1 Department of Biochemistry, College of Pure and Applied Sciences, Landmark University, P.M.B 1001, Omu Aran 251101, Nigeria; 2 Department of Chemical Sciences, Faculty of Science and Science Education, Anchor University, P.M.B 001, Ipaja, Nigeria

**Keywords:** antioxidant enzymes, monosodium glutamate, reactive oxygen species, reproductive dysfunction, sperm quality, testosterone

## Abstract

Reproductive dysfunction is often characterized by malfunction of the reproductive tissues, which may lead to disruption of the synergistic rhythm that should bring about a progression of sexual events and the conception of new life. This may therefore result in the sexual dysfunction and infertility that can be seen in couples having prolonged biological difficulty in reproducing their offspring after having unrestricted sexual intercourse for at least twelve months. Several factors have been implicated in the cause and progression of reproductive dysfunction, including poor nutrition, drug side effects, disease states, and toxicant ingestion. A well-known food additive that has been found to be potent at initiating reproductive anomalies in males is monosodium glutamate (MSG). This regular flavor enhancer is widely used as a taste enhancer in several diets. The different mechanisms by which it may induce reproductive dysfunctions include spermatogenic alteration resulting in a low sperm count, high sperm abnormality, reduced live sperm and decreased sperm pH, oxidative damage (increased lipid peroxidation and reduced antioxidant enzyme activities), histological alteration (blood hemorrhage, distorted germ and Sertoli cells), as well as gonadotropin imbalance (reduced testosterone, luteinizing hormone, and follicle-stimulating hormone concentrations). Therefore, this review discusses various established mechanisms through which MSG may induce reproductive dysfunction and the treatment strategies to ameliorate its toxic effects.

## 1. Introduction

The adult male reproductive system consists of two testes, each joined to its own epididymis and connected to the penis via the vas deferens, and functioning majorly in the production and transportation of sperm for the fertilization of an ovum, leading to the development of an offspring [1]. Germ cells develop in the testes and travel through the epididymis (caput to cauda) where they mature and gain motility [2]. During copulation, sperm is released as semen into the female reproductive tract, where the final stages of maturation takes place (capacitation) and leave the sperm ready for fertilization should an ovum be present [3].

Male reproductive dysfunction describes a condition where one or more of the components of the male reproductive system is malfunctioning or performs below its expected capability. This may have a debilitating effect on the individual and may result in other secondary conditions [4]. Some of the implicated factors for male reproductive dysfunction include hormonal disorders, reactive oxygen species, testicular inflammation, endocrinal disturbance, genital infection, heat, smoking and alcohol, illness, injury, chronic health problems, heavy metals, genetic defects, exposure to radiation, lifestyle, and diet [5].

The German chemist Karl Heinrich Ritthausen discovered monosodium glutamate. MSG is a subset of glutamate which is an important but “non-essential” amino acid found in several foods, including beef, milk, tuna, and vegetables, and plays an important role in human metabolism [6]. MSG is formulated from water, glutamate, and sodium, and it is a major food flavor enhancer, which serves to exaggerate the inherent flavor of foods. The induction of myriad undesirable conditions such as weakness, numbness, muscle pain, headaches, dizziness, and flushing have been associated with MSG consumption [7]. Pre-clinical studies revealed that repeated MSG ingestion may also trigger asthma, cancer-induced obesity, diabetes, and oxidative stress. Toxicities such as hepatotoxicity, genotoxicity, reproductive toxicity, and renal toxicity, as well as neurotoxic effects have also been reported to accompany MSG intake. MSG ingestion has also been linked with Parkinson’s disease, Alzheimer’s disease, addiction, brain trauma, anxiety, stroke, depression, and epilepsy [6,8]. MSG has also been associated with male reproductive dysfunction by triggering a hemorrhage in the testis as well as deterioration of sperm cell structure and production [9]. Glutamate, a major component of MSG, is present in large quantities in the body and is sourced from dietary protein or food containing free glutamate (MSG/hydrolyzed protein). Glutamate is metabolized mainly in the small intestine, where other amino acids (aspartate and glutamine) are catabolized. It is a major substrate for the synthesis of protein, as it is contained in 20–40% of most proteins. Excitatory amino acid carrier 1 (EAAC-1) (intestine), glutamate/aspartate transporter-1 (GLAST-1), and glutamate transporter-1 (GLT-1) (stomach) respectively, are the major glutamate and glutamine active transporters. These transporters are dependent on sodium ion concentration, and can be inhibited competitively [9].

Glutamate can be metabolized into free amino acids absorbed into the gut for further breakdown [7]. The metabolized products include α-ketoglutarate via transamination (alanine transferase and aspartate transferase) and deamination using glutamate dehydrogenase, glutamine substrate through glutamine synthetase, and precursors for glutathione and N-acetylglutamine generation. The product, α-ketoglutarate, enters the tricarboxylic acid (TCA) cycle in the mitochondrial matrix for the production of energy and release of CO_2_. Therefore, increased glutamate in the diet could increase energy generation by increasing the level of transamination and deamination, conversion of amino acids into glucose (gluconeogenesis), and conversion to other products like glutathione, GABA, N-acetylglutamate, and γ-carboxyglutamine [8].

Neurotransmitters are chemical messengers carrying information between nerve cells to influence each other in the central nervous system (CNS). Neurotransmitters are found at the terminal buttons of transmitting nerves and they inhibit or excite cell targets. Glutamate is a major excitatory neurotransmitter, which enables the rapid transmission of synapses. It is also a precursor to γ-butyric acid (GABA), another important neurotransmitter. Memory, cognition, and sensation (taste, hearing, and sight) are the consequences of glutamate neurotransmission. Glutamate also has a trophic role in developing the CNS and so controls reproduction, movement, and the survival of neuronal progenitors [8,9].

## 2. MSG and the Induction of Reproductive Dysfunction

Several investigations have documented the induction of different modes of reproductive injury by MSG in laboratory animals. These studies are summarized in Table 1. A few other studies have also documented the treatment of these MSG-induced alterations with natural products (Table 2). 

## 3. Mechanism of MSG-Induced Testicular Alteration

**Figure 1:**
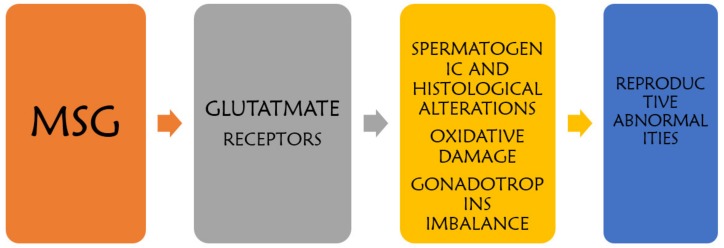
Mechanism of MSG-induced testicular alterations.

The action of MSG on the male reproductive morphology and function may be as a result of its diverse influence on cells, thereby initiating spermatogenic alterations, oxidative damage, histological alteration, and gonadotropin imbalance which may eventually culminate into reproductive abnormalities in the males as shown in Figure 1.

There seems to be a scarcity of empirical data on the evaluation of MSG intake on human male reproductive functions; mostly, available animal model studies are extrapolated to humans.

### 3.1. Oxidative Stress

The organs of the reproductive system are targets of reactive oxygen species (ROS) because of adipose tissue present in these organs [13]. Studies have revealed an increase in the lipid peroxidation (malondialdehyde, MDA) and decreased antioxidant activity (reduced glutathione, GSH) as well as noticeable increase in testicular oxidative stress and a corresponding reduction in antioxidant/antioxidant enzyme activities after MSG administration [7,13,19]. Increased production of free radicals caused by MSG could lead to lipid peroxidation and sperm membrane dysfunction, sperm DNA damage, and impaired sperm movement. The abundance of unsaturated fat (plasma membrane) and low levels of antioxidants (cytoplasm) make the testes and sperm cells susceptible to oxidative stress [20]. Patients with asthenozoospermia were found to have a high ROS generation in seminal plasma as well as sperm membrane damage mediated by MSG [7,19]. The direct implication of this ROS-induced damage on membrane integrity are impaired sperm motility and viability [20]. Therefore, therapeutic agents such as antioxidants may be useful in reversing MSG-induced reproductive toxicity. 

### 3.2. Neurotoxicity

The neurotoxic effect of MSG causes excitotoxicity in the brain through disruption of the hypothalamic–pituitary-axis pathway (HPA) [13]. Glutamate is an excitatory neurotransmitter, and a high influx of neuron intracellular calcium caused by high glutamate may lead to neuronal death. HPA disruption may reduce levels of sex hormones, including testosterone, follicle-stimulating hormone, and luteinizing hormone. This ultimately leads to alterations in sperm quality [21]. Spermatogenesis is totally dependent on the sex hormones and androgen-dependent organs of the reproductive system, which include the prostate gland, epididymis and seminal vesicles. Any androgen hormone (i.e., testosterone, luteinizing hormone, and follicle-stimulating hormone) disorder will therefore have a negative impact on the reproductive tissues [7].

### 3.3. Histomorphological Alterations

Alterations of the testicular histopathology such as spermatogenic arrest, low sperm production, and edema have previously been reported [7,10,11,13]. Meanwhile, another study observed no overt histopathological changes in the MSG-treated animals [15]. Low spermatogonia levels have been linked with maturation arrest in MSG-exposed animals, and this correlates with a low level of testosterone leading to inhibition of spermatogenesis [7,10,11,12,14,15,16]. Other studies, however, observed improved testicular histopathology after the administration of selenium, vitamin E, and curcumin, respectively [7,22]. Treatments such as graviola extract, vitamin C, vitamin E, camel milk, propolis, quince extract, and curcumin have proven to provide protective effects against MSG-induced histomorphological testicular toxicities [17,18,19,20,21,22].

### 3.4. Glutamate Receptor Dysfunction

Another mechanism of MSG-induced male reproductive toxicity is via glutamate receptors, as MSG directly affects the glutamate transporter on the epithelium of seminiferous tubules. Glutamate receptors are found in different organs and tissues, including the endocrine glands, hypothalamus, thymus, ovaries, liver, kidney, and testis. The testis has been found to exhibit morphological alterations subsequent to MSG treatment due to mal-expression of glutamate receptor in the testis [7,10,21].

### 3.5. Brief Clinically Observed Adverse Effect of MSG

Clinical trials conducted in the past have revealed the interplay between MSG and hunger and food intake. In one study, 32 volunteers were screened for the effect of MSG on food intake. It was observed that those who consumed soup containing MSG had increased hunger and food intake when compared to those who took soup without MSG [8,23]. In another study involving 100 French men given an MSG-added diet, an obvious increase in food intake was noticed [8,24]. There have been clinical reports on the direct relationship between MSG intake and obesity in humans [8,25,26,27]. Clinical trials have also shown that the consumption of MSG could result in certain allergic reactions in humans [8].

## 4. Conclusions

MSG may induce male reproductive toxicity via different mechanisms (e.g., oxidative damage, histomorphological alterations, hormonal dysfunction, and reduced sperm quality). Further studies should be focused on providing a more comprehensive mechanism for MSG-induced reproductive dysfunctions in order to generate a better management strategy for the condition. Clinical studies on human subjects to evaluate the adverse effects of MSG on the male reproductive system are also advocated.

## Figures and Tables

**Table 1:** Reproductive injury induced by monosodium glutamate (MSG) administration to laboratory animals.

Serial number	MSG Induced Alteration	Animal Type and Average Body Weight	Days of Administration and Type	Number of Animals and Dosages	Authors
1	Increased body weight; decreased sperm concentration, motility, and viability; Alteration in testis histological structure; Seminiferous tubules deterioration.	Albino rats 150 g	90 days Oral	20 rats: 4 (5/group) * MSG 1: 0 mg/kg * MSG 2: 35 mg/kg * MSG 3: 350 mg/kg * MSG 4: 500 mg/kg	[10]
2	Testicular histological changes in germinal epithelium and Leydig cells (hypertrophied); Fewer spermatogenic cells in tubules; Low serum LH and testosterone levels.	Neonate	75 days Subcutaneous	14 mice Control: distilled water (DW) * MSG: (2 mg/kg)	[11]
3	Decrease in weight of testis, epididymis, seminal vesicle and prostate; Decreased hormone levels: testosterone and follicle-stimulating hormone; Reduced sperm counts.	Wistar rats 180 g	120 days Subcutaneous	18 rats: 2(9) (Control: 2.0% NaCl) * (MSG: 4.0 mg/kg)	[12]
4	Increased SOD level in epididymis, prostate gland, and seminal vesicle; Reduced GSH level in the organs; Increased lipid peroxidation (MDA) and protein; oxidation in the organs; Histopathological alteration in organs.	Sprague Dawley Rats 170–200 g	28 days Oral	24 rats: 3 (8/group) Control:1 mg/kg DW * MSG 1: 60 mg/kg * MSG 2: 120 mg/kg	[13]
5	Testicular morphological alteration; Decreased testosterone level; Low sperm count.	Sprague Dawley male rats	30 days (twice a day)	32 rats: 4 (8/group) Control: DW MSG 1: 0.25 g/kg MSG 2: 3 g/kg * MSG 3: 6 g/kg	[14]
6	Decreased epididymal sperm count; Reduced serum testosterone level; No overt histopathological lesion.	Sprague Dawley rats 160–180g	Oral 42 days (every 48 h)	28 rats: 4 (7/group) * MSG 1: 0 mg/kg * MSG 2: 1 mg/kg * MSG 3: 2 mg/kg * MSG 4: 4 mg/kg	[15]
7	Decrease in sperm count and abnormal sperm morphology; Decrease in testicular weight and tubular diameter; Reduction in germinal epithelium height.	Wistar male rats	14 days intraperitoneal	24 rats: 4 (6/group) Control group * Experimental group: 4 mL/kg	[16]

* Groups showing the observed effects. GSH: reduced glutathione; MDA: malondialdehyde; SOD: superoxide dismutase.

**Table 2:** Treatment options to ameliorate MSG-induced reproductive tissue alterations.

Serial number	Effect of MSG and Treatment	Animals Used and Body Weight	Days of Administration and Type	Number of Rats and Dosages	Authors
1	Histological alterations (blood hemorrhage, distorted germ cells, and few Sertoli cells) Damaged seminiferous tubules Treatment with graviola extract (GE) Improved seminiferous tubules and germ cell Restored testis configuration	Albino rats 120–150 g	4–8 weeks Oral	48 rats: 4 (12/group) Group 1: distilled water Group 2: GE (100 mg/kg) Group 3: MSG (4 mg/kg) Group 4: MSG (4 weeks) + GE (4 weeks)	[17]
2	Reduced testis and epididymis weight Reduced sperm motility, count, and viability Treatment with vitamin C Restored MSG effect on organ weight Repaired sperm characteristics	Albino rats	65 days Oral: Vit C Intraperitoneal: MSG	30 rats: 5(6/group) Control: DW Group 2: Vit. C (100 mg/kg) Group 3: MSG (2 mg/kg) Group 4: MSG + Vit C Group 5: 2 MSG + Vit C	[18]
3	Reduced testicular antioxidant activities Increased lipid peroxidation Low testosterone and luteinizing hormones Sperm motility and abnormalities Downregulation of steroidogenesis genes Treatment with camel milk and vitamin E Restored antioxidant and hormone levels Upregulated genes and restored sperm analysis	Wistar rats 170–200 g	28 days Oral	40 rats: 4 (10/group) Control: DW MSG (2 g/kg) MSG + Vit E (20 mg/kg) MSG + camel milk (166.6 mL/24 h/10 rats)	[19]
4	Increased oxidative stress (MDA) Reduced antioxidant activity (catalase, superoxide dismutase and glutathione peroxidase) Altered histology of the testis Treatment with vitamin E and selenium Ameliorated MSG effect on lipid peroxidation and antioxidant activities Restored testicular histological structures	Wistar rats 150–200 g	30 days Oral	120: 12 (10) rats Control:1 mg corn oil MSG: 6 mg/kg MSG: 17.5 mg/kg MSG: 60 mg/kg Vit. E: 150 mg/kg Vit. E: 200 mg/kg Selenium (Se): 0.25 mg/kg Se: 1.0 mg/kg MSG 60 mg/kg + Vit E 150 mg/kg MSG 60 mg/kg + Vit E 200 mg/kg MSG 60 mg/kg + Se 0.25 mg/kg MSG 60 mg/kg + Se 1.0 mg/kg	[7]
5	Decreased body and testis weight Decreased testosterone and semen quality Treatment with propolis Increased body and testis weight Elevated testosterone and semen quality	Rabbits	12 weeks Oral	20 rats: 4 (5/group) Group 1: control Group 2: propolis (50 mg/kg) Group 3: MSG (8 mg/kg) Group 4: propolis + MSG	[20]
6	Low testosterone and follicle-stimulating hormone (FSH) level Reduced epididymal sperm concentration No change in the luteinizing hormone (LH) Treatment with quince leaf extract Improvement in testosterone and FSH level Reduced sperm motility induced by MSG	Wistar rats 120 ± 20 g	8 weeks Intraperitoneal	60 rats: 6 (10/group) Control: no treatment MSG 30 mg/kg MSG 60 mg/kg MSG 30 mg/kg + quince extract (QE) 500 mg/kg MSG 60mg + QE 500 mg/kg QE 500 mg/kg	[21]
7	Decreased testis weight and sperm count Histological alteration and reduced hormone Spermatogenic loss and deformed Sertoli cells Treatment with curcumin Improved histopathological alteration Increased sperm count and sex hormones	Sprague Dawley rats 140 ± 5 g	Oral	65 rats in total Control: 10 rats Group 2: 15 rats—150 mg/kg curcumin Group 3: 20 rats—4 mg/kg MSG Group 4: 20 rats—MSG + curcumin	[22]
